# Impaired Autophagy Induced by oxLDL/*β*2GPI/anti-*β*2GPI Complex through PI3K/AKT/mTOR and eNOS Signaling Pathways Contributes to Endothelial Cell Dysfunction

**DOI:** 10.1155/2021/6662225

**Published:** 2021-06-14

**Authors:** Guiting Zhang, Chao He, Qianqian Wu, Guoying Xu, Ming Kuang, Ting Wang, Liangjie Xu, Hong Zhou, Wei Yuan

**Affiliations:** ^1^Department of Cardiology, Affiliated Hospital of Jiangsu University, 438 Jiefang Road, Zhenjiang, Jiangsu 212013, China; ^2^Department of Clinical Laboratory and Hematology, School of Medicine, Jiangsu University, 301 Xuefu Road, Zhenjiang, Jiangsu 212013, China; ^3^School of Medical Technology, Jiangsu College of Nursing, 9 Keji Avenue, Huaian, Jiangsu 223007, China

## Abstract

Endothelial cell dysfunction plays a fundamental role in the pathogenesis of atherosclerosis (AS), and endothelial autophagy has protective effects on the development of AS. Our previous study had shown that oxidized low-density lipoprotein/*β*2-glycoprotein I/anti-*β*2-glycoprotein I antibody (oxLDL/*β*2GPI/anti-*β*2GPI) complex could promote the expressions of inflammatory cytokines and enhance the adhesion of leukocytes to endothelial cells. In the present study, we aimed to assess the effects of oxLDL/*β*2GPI/anti-*β*2GPI complex on endothelial autophagy and explore the associated potential mechanisms. Human umbilical vein endothelial cells (HUVECs) and mouse brain endothelial cell line (bEnd.3) were used as models of the vascular endothelial cells. Autophagy was evaluated by examining the expressions of autophagic proteins using western blotting analysis, autophagosome accumulation using transmission electron microscopy, and RFP-GFP-LC3 adenoviral transfection and autophagic flux using lysosome inhibitor chloroquine. The expressions of phospho-PI3K, phospho-AKT, phospho-mTOR, and phospho-eNOS were determined by western blotting analysis. 3-Methyladenine (3-MA) and rapamycin were used to determine the role of autophagy in oxLDL/*β*2GPI/anti-*β*2GPI complex-induced endothelial cell dysfunction. We showed that oxLDL/*β*2GPI/anti-*β*2GPI complex suppressed the autophagy, evidenced by an increase in p62 protein, a decrease in LC3-II and Beclin1, and a reduction of autophagosome generation in endothelial cells. Moreover, inhibition of autophagy was associated with PI3K/AKT/mTOR and eNOS signaling pathways. Rapamycin attenuated oxLDL/*β*2GPI/anti-*β*2GPI complex-induced endothelial inflammation, oxidative stress, and apoptosis, whereas 3-MA alone induced the endothelial injury. Our results suggested that oxLDL/*β*2GPI/anti-*β*2GPI complex inhibited endothelial autophagy via PI3K/AKT/mTOR and eNOS signaling pathways and further contributed to endothelial cell dysfunction. Collectively, our findings provided a novel mechanism for vascular endothelial injury in AS patients with an antiphospholipid syndrome (APS) background.

## 1. Introduction

As a complex and chronic progressive vessel disease, atherosclerosis (AS) is characterized by early endothelial dysfunction, and it leads to the morbidity of dysfunctional cardiovascular events worldwide [1–3]. Endothelial cell dysfunction plays a fundamental role in the pathogenesis of AS, resulting in the initiation of AS and the formation of atherosclerotic plaques [4]. The pathophysiological process of endothelial dysfunction is multifactorial and complex, and such process includes increased oxidative stress, abnormal autophagy, apoptosis, and inflammation [5–8].

Autophagy is an important biological process for lysosomal self-digestion, in which protein aggregates and damaged organelles are engulfed by double-membraned autophagosomes and transported to the lysosomes for degradation, thus, contributing to the maintenance of normal cellular function and survival [9–11]. Increased expressions of autophagy-degrading substrate p62, autophagy constituent protein LC3-II, and autophagy-related protein Beclin1 reflect the induction of autophagy [12]. The term autophagic flux represents the whole autophagic procedure, including autophagosome formation, transport of the autophagic substrate to lysosomes, and autophagosomal degradation in lysosomes [10]. The autophagic flux can be evaluated using inhibitors of autophagosome-lysosome fusion, such as chloroquine (CQ) and hydroxychloroquine (HCQ) [13]. Meanwhile, several molecular and cell signaling pathways have been implicated in regulating autophagy, such as PI3K/AKT/mTOR and AKT/eNOS pathways [14–16]. Under stressful conditions, such as starvation, hypoxia, and nutrient deficiency, the PI3K/AKT signaling pathway negatively regulates autophagy by mediating the mTOR expression in endothelial cells [17, 18]. Moreover, eNOS is another classic downstream target of AKT, and the AKT/eNOS signaling pathway is involved in the regulation of NO production and autophagy [16, 19].

Accumulating evidence shows that proper autophagy has protective effects on the development of AS by participating in the regulation of cellular injury in endothelial cells and vascular smooth muscle cells (VSMCs) [20–23], although the underlying molecular mechanism remains largely unexplored. Previous experiments suggest that adequate endothelial autophagic flux limits the formation of atherosclerotic plaques by preventing endothelial apoptosis, senescence, and inflammation [5]. On the one hand, excessive activation of autophagy can result in endothelial cell death and plaque destabilization [24, 25]. On the other hand, autophagy deficiency has been reported to increase the endothelial inflammation in patients with nonalcoholic steatohepatitis [26] and to accelerate the formation of atherosclerotic plaques in mice [22].

Antiphospholipid syndrome (APS) is an autoimmune disease characterized by thrombosis, pregnancy loss, and the presence of antiphospholipid antibodies (aPL), anti-*β*2 glycoprotein I antibodies (anti-*β*2GPI), and lupus anticoagulant (LA) [27, 28]. *β*2GPI, which can bind to oxidized low-density lipoproteins (oxLDL) via domain V, is accepted as a potential autoantigen to accelerate the process of AS in patients with an APS background [29]. Based on these studies, it is hypothesized that oxLDL/*β*2GPI/anti-*β*2GPI complex, the combination of the oxLDL/*β*2GPI complex and anti-*β*2GPI, is a circulating immune complex that exerts a proatherogenic effect in patients with APS, which has been validated by published studies [30–32]. In previous studies, we demonstrated that oxLDL/*β*2GPI/anti-*β*2GPI complex could induce the foam cell formation of macrophages and VSMCs and the expressions of inflammatory cytokines in endothelial cells, resulting in the formation of atheromatous plaques with an APS background [33–35]. However, little evidence is available about the relationship between oxLDL/*β*2GPI/anti-*β*2GPI complex and autophagy in endothelial cells. In the present study, we investigated the effects of autophagy on oxLDL/*β*2GPI/anti-*β*2GPI complex-induced endothelial dysfunction and its underlying molecular mechanisms.

## 2. Materials and Methods

### 2.1. Cell Culture and Treatment

HUVECs and bEnd.3 cells were purchased from the Shanghai Institutes for Biological Sciences. The cells were maintained in Dulbecco's Modified Eagle Medium (DMEM, Biological Industries, Israel) supplemented with 10% heat-inactivated fetal bovine serum (FBS, Biological Industries, Israel), 4.5 g/L glucose, 1% glutamine, and 1% penicillin/streptomycin (Gibco, USA) at 37°C in a humidified atmosphere containing 5% CO_2_. All experiments were carried out when the cell density was 90–100%.

Cells were stimulated with oxLDL (50 *μ*g/mL) (Yiyuan Biotech, Guangzhou, China), oxLDL (50 *μ*g/mL)/*β*2GPI (100 *μ*g/mL) (Sigma-Aldrich, USA), oxLDL (50 *μ*g/mL)/anti-*β*2GPI (100 *μ*g/mL) (Sigma-Aldrich; USA), *β*2GPI (100 *μ*g/mL)/anti-*β*2GPI (100 *μ*g/mL), or oxLDL (50 *μ*g/mL)/*β*2GPI (100 *μ*g/mL)/anti-*β*2GPI (100 *μ*g/mL) for the indicated times. The DMEM (with 10% FBS) was employed as the blank control in the current study. For preparation of the complex of oxLDL/*β*2GPI, 50 *μ*g oxLDL and 100 *μ*g *β*2GPI were added to 1 mL DMEM (with 10% FBS) and then incubated at 37°C and pH 7.4 for 16 h. The complex of oxLDL/anti-*β*2GPI, *β*2GPI/anti-*β*2GPI, and oxLDL/*β*2GPI/anti-*β*2GPI were prepared by incubating oxLDL (50 *μ*g/mL), *β*2GPI (100 *μ*g/mL), or oxLDL (50 *μ*g/mL)/*β*2GPI (100 *μ*g/mL) complex with anti-*β*2GPI (100 *μ*g/mL) at 37°C for 30 min. The preparation and identification of the abovementioned reagents were determined by preliminary experiments and previous studies [35, 36].

For the inhibition of the PI3K/AKT/mTOR pathway and eNOS pathway, the cells were pretreated with 10 *μ*M PI3K inhibitor LY294002 (Sigma-Aldrich; USA), 1 *μ*M AKT inhibitor AZD5363 (Abmole, USA), 1 *μ*M mTOR inhibitor rapamycin (Abmole, USA), or 100 *μ*M eNOS inhibitor L-NAME (Abmole, USA) for 4 h, followed by incubation in the presence or absence of oxLDL/*β*2GPI/anti-*β*2GPI complex for 24 h. Cells were pretreated with 10 *μ*M CQ (Sigma-Aldrich, USA) for 4 h, followed by incubation in the presence or absence of oxLDL/*β*2GPI/anti-*β*2GPI complex for an additional 24 h. For the inhibition or activation of autophagy, the cells were treated with 5 mM 3-methyladenine (3-MA) (Abmole, USA) or 1 *μ*M rapamycin in the presence or absence of oxLDL/*β*2GPI/anti-*β*2GPI complex for 24 h.

### 2.2. Western Blotting Analysis

Total cellular protein was extracted using RIPA lysis buffer (Beyotime Institute of Biotechnology, Shanghai, China) supplemented with 1% phenylmethanesulfonyl fluoride (PMSF) (Aksmoics, Shanghai, China) and 1% phosphatase inhibitor (Aksmoics, Shanghai, China). Protein concentration was determined by the BCA Protein Assay Kit (Beyotime Institute of Biotechnology). Briefly, equal amounts of proteins (100 *μ*g) were subjected to sodium dodecyl sulfate-polyacrylamide gel electrophoresis (SDS-PAGE) using 10% or 12% gels and transferred onto polyvinylidene difluoride (PVDF) membranes (Millipore, Bedford, MA, USA). The blotted membranes were first probed with primary antibodies against Beclin1, LC3, p62, ICAM-1 (all above four antibodies: 1:  1,000; Cell Signaling Technology, MA, USA), phosphoinositide 3-kinase (PI3K) and phosphate-PI3K (Tyr458) (1:  1,000; Cell Signaling Technology), AKT and phosphate-AKT (Ser473) (1:  1,000; Cell Signaling Technology), mammalian target of rapamycin (mTOR) and phosphate-mTOR (Ser2448) (1:  1,000; Cell Signaling Technology), eNOS (Wanlei Biotech, China) and phosphate-eNOS (Ser 1177) (1 : 1000, Abcam, Cambridge, UK), and *β*-actin (1 : 5,000, Bioworld, Nanjing, China) at 4°C overnight, followed by incubation with horseradish peroxidase-conjugated secondary antibody (1 : 5,000; Bioworld) at room temperature (RT) for 1 h. Immunoreactive bands were visualized using enhanced ECL western blotting detection reagent (Vazyme, Nanjing, China) on an Image Quant LAS 4000 imager, and the densitometric analysis was performed using LANE 1D (Beijing Sage Creation Science Co., Ltd.).

### 2.3. Transmission Electron Microscopy (TEM)

HUVECs and bEnd.3 cells were fixed with 2.5% glutaraldehyde in phosphate buffer and stored at 4°C overnight. The cells were further fixed with 1% osmium tetroxide and stained with 1% uranyl acetate, followed by a gradient dehydration step using ethanol at concentrations of 50%, 70%, 95%, and 100%. The samples were then embedded in epoxy resin. After placing 100 nm sections on a copper mesh, the materials were analyzed using a transmission electron microscope (HITACHI-HT7700, Japan).

### 2.4. mRFP-GFP-LC3 Adenoviral Transfection

HUVECs and bEnd.3 cells were seeded into 24-well plates at a density of 1.0 × 10^5^ cells/well, and mRFP-GFP-LC3 adenovirus (HanhengBio Technology, Shanghai, China) with a multiplicity of infection (MOI) value of 50 was loaded according to the manufacturer's instructions. Subsequently, the cells were subjected to the abovementioned treatments. The cells were washed with PBS three times and fixed with a mounting medium (Solarbio, Beijing, China). Fluorescent images were acquired using a confocal laser scanning microscope equipped with a 60× objective lens (LSM 880 with Airyscan; Zeiss, Dublin, CA, USA).

### 2.5. Reverse Transcription-Quantitative Polymerase Chain Reaction (RT-qPCR)

Total RNA was extracted from cells using TRIzol® reagent (Invitrogen; USA). Complementary DNA (cDNA) was synthesized using the HiScriptTM First-strand cDNA Synthesis Kit (Vazyme, China). RT-qPCR was performed using SYBR Green I dye (Vazyme, China) and 10 ng cDNA. Briefly, after an initial denaturation step at 95°C for 30 s, the amplifications were carried out with 39 cycles at a melting temperature of 95°C for 30 s, an annealing temperature of 58°C (ICAM-1, IL-6, and *β*-actin)/54°C (IL-1*β*) for 30 s, and an extension temperature of 72°C for 30 s. The primer sequences are shown in Table S1, and *β*-actin was selected as the housekeeping gene. The expressions of target genes were calculated using the 2^-*Δ*∆Ct^ method.

### 2.6. Enzyme-Linked Immunosorbent Assay (ELISA)

HUVECs were seeded into 24-well plates at a density of 1.0 × 10^5^ cells/well and treated with different stimuli as above described. The concentrations of IL-1*β* and IL-6 in the cell culture supernatants were analyzed using ELISA kits for IL-1*β* (Multiscience, China) and IL-6 (Multiscience, China) according to the manufacturers' instructions. The concentrations of the cytokines were expressed as pg/mL.

### 2.7. Intracellular Reactive Oxygen Species (ROS) Detection

ROS was measured using the 2′, 7′-dichlorodihydrofluorescein diacetate (DCFH-DA) probe according to the manufacturer's instructions. Cells were incubated with 10 *μ*M DCFH-DA (Jiancheng, Nanjing, China) at 37°C for 30 min. Cell images were captured by the BioTekCytation 5 Cell Imaging Multi-Mode Reader (BioTek, USA). Besides, the mean fluorescence of labeled cells was determined by flow cytometry (BD Biosciences, USA), with an excitation at 488 nm and emission at 530 nm. The data were analyzed using FlowJo software (version 10.0.7).

### 2.8. Intracellular Superoxide Dismutase (SOD) Detection

The activity of SOD in cells was examined using the xanthine oxidase method provided by the standard assay kit (Jiancheng, Nanjing, China). After stimulation, the total protein was collected using the supersonic schizolysis method, and the protein concentration was determined by a BCA Protein Assay Kit (Beyotime, Hangzhou, China). Samples were then determined according to the manufacturer's instructions. The values were expressed as units per mg protein, where one unit was defined as the amount of SOD inhibiting the reaction rate by 50% at 25°C.

### 2.9. Annexin V-FITC/PI Apoptosis Detection

The apoptotic rate of HUVECs was detected using an AnnexinV–fluorescein isothiocyanate (FITC) Apoptosis Detection Kit with propidium iodide (PI) (BD Biosciences, CA, USA) according to the manufacturer's instructions. After stimulation, the cells were digested with trypsin and suspended in 1× binding buffer at a density of 1 × 10^6^ cells/mL. Cells were then stained with FITC annexin V and PI at room temperature for 15 min. Finally, samples were detected using flow cytometry (BD Biosciences, USA), and the total apoptotic rates (*Q*2 + *Q*3) were calculated. The data were analyzed using FlowJo software (version 10.0.7).

### 2.10. Statistical Analysis

All data were expressed as the mean ± SEM. Differences between the control and experimental conditions were assessed using the one-way ANOVA, followed by Tukey's multiple group comparison test. Two-factor treatment results were analyzed by two-way ANOVA with Tukey's test. Statistical analyses were performed with GraphPad Prism (version 7.0.0). Significant differences between the two groups were indicated by either an asterisk ∗ or ns, where ns represents nonsignificance, ∗ represents *P* < 0.05; ∗∗ represents *P* < 0.01; ∗∗∗ represents *P* < 0.001, and ∗∗∗∗ represents *P* < 0.0001. All experiments were independently performed at least three times.

## 3. Results

### 3.1. Effects of oxLDL/*β*2GPI/anti-*β*2GPI Complex on Autophagy of Endothelial Cells

**Figure 2:**
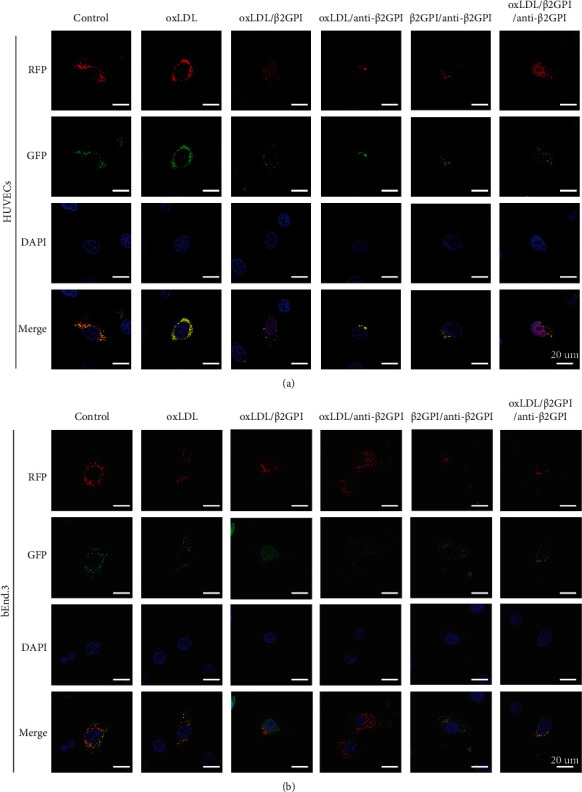
RFP-GFP-LC3 tandem fluorescent probe analysis of the effect of oxLDL/*β*2GPI/anti-*β*2GPI complex on endothelial cell autophagy. HUVECs and bEnd.3 cells were incubated with oxLDL, oxLDL/*β*2GPI, oxLDL/anti-*β*2GPI, *β*2GPI/anti-*β*2GPI, and oxLDL/*β*2GPI/anti-*β*2GPI complex for 24 h. Representative images (magnification, ×600) of RFP-GFP-LC3 puncta in HUVECs (a) and bEnd.3 cells (b). Yellow puncta (RFP^+^ and GFP^+^) represents autophagosomes, and red puncta (RFP^+^ and GFP^−^) represents autolysosomes. Scale bar: 20 *μ*m. All tests were conducted at 5 times (*n* = 5), and a representative image was shown.

We used HUVECs and bEnd.3 cells as endothelial cell models to evaluate the effects of oxLDL/*β*2GPI/anti-*β*2GPI complex on the autophagy of vascular endothelial cells. By western blotting analysis, we determined the autophagy level. Changes were most evident at 24 h in HUVECs treated with oxLDL/*β*2GPI/anti-*β*2GPI complex, and therefore, this time point was selected to be the optimum incubation time in subsequent experiments (Figure S1). Then, we found that the expressions of Beclin-1 and LC3-II were significantly downregulated, while the expression of p62 was significantly upregulated in HUVECs (*P* < 0 : 05; Figure 1(a)) and bEnd.3 cells (*P* < 0 : 05; Figure 1(b)) in the oxLDL/*β*2GPI/anti-*β*2GPI complex group compared with the DMEM control group and oxLDL group. The aggregation of LC3 puncta was markedly decreased in the oxLDL/*β*2GPI/anti-*β*2GPI complex group compared with the DMEM control group and oxLDL group (Figure 2). Besides, the oxLDL group showed markedly increased expressions of autophagic proteins and aggregation of LC3 puncta compared with the DMEM control group (*P* < 0 : 05; Figures 1(a) and 1(b)) (Figure 2). TEM demonstrated that the mean number of autophagosomes per cell was significantly decreased in the oxLDL/*β*2GPI/anti-*β*2GPI complex group compared with the DMEM control group (*P* < 0 : 01; Figures 1(c) and 1(d)).

### 3.2. Effects of oxLDL/*β*2GPI/anti-*β*2GPI Complex on the Autophagic Flux in Endothelial Cells

**Figure 3:**
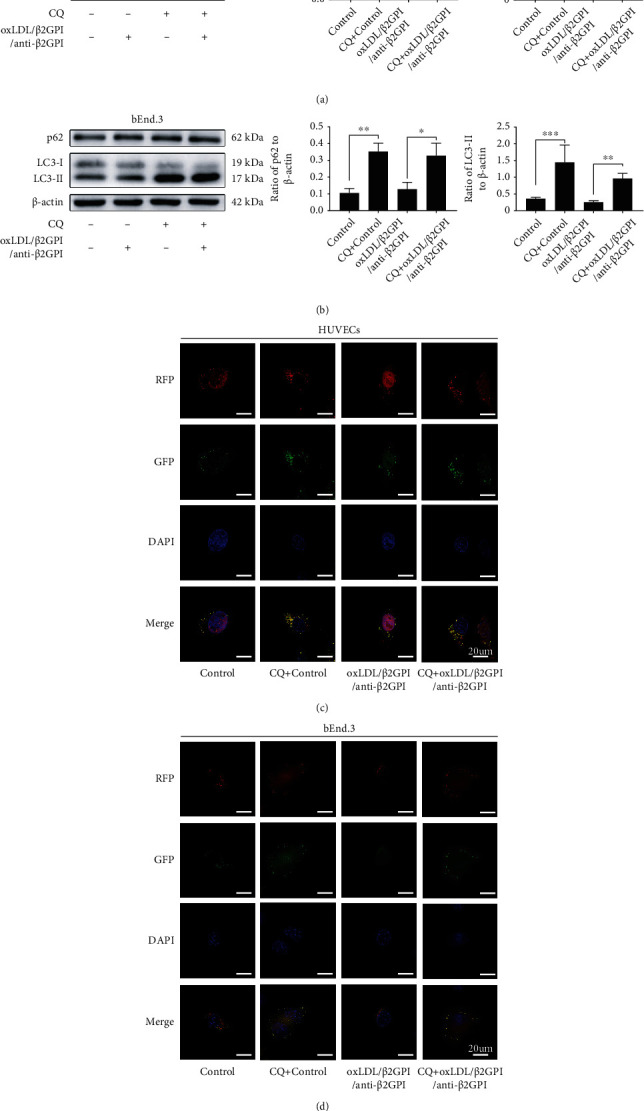
OxLDL/*β*2GPI/anti-*β*2GPI complex disrupts the autophagic flux in endothelial cells. HUVECs and bEnd.3 cells were incubated in the presence or absence of oxLDL/*β*2GPI/anti-*β*2GPI complex for 24 h. CQ (1 *μ*M), a lysosome inhibitor, was added 4 h before oxLDL/*β*2GPI/anti-*β*2GPI complex treatment. Western blotting analysis and quantification of p62 and LC3-IIin HUVECs (a) and bEnd.3 cells (b). Representative images (magnification, ×600) of RFP-GFP-LC3 puncta in HUVECs (c) and bEnd.3 cells (d). Yellow puncta (RFP^+^ and GFP^+^) represents autophagosomes, and red puncta (RFP^+^ and GFP^−^) represents autolysosomes. Scale bar: 20 *μ*m. ^∗^*P* < 0.05, ^∗∗^*P* < 0.01, ^∗∗∗^*P* < 0.001, and ^∗∗∗∗^*P* < 0.0001 indicate statistically significant differences. ns: nonsignificant differences. All values are denoted as means ± SD from three independent experiments (*n* = 3), and a representative blot/image was shown.

To evaluate the effects of oxLDL/*β*2GPI/anti-*β*2GPI complex on autophagic flux in endothelial cells, the cells were pretreated with CQ (autophagy inhibitor) and incubated in the presence or absence of oxLDL/*β*2GPI/anti-*β*2GP I complex for 24 h. We found that CQ pretreatment had significant promotion effects on the accumulation of p62 and the expression of LC3-II compared with the control group, reflecting impairment in the autophagic flux (*P* < 0 : 01; Figures 3(a) and 3(b)). This effect was similar to the aggregation of LC3 puncta induced by CQ (Figures 3(c) and 3(d)). Moreover, CQ pretreatment further promoted the accumulation of p62 and significantly increased the expression of LC3-II compared with the oxLDL/*β*2GPI/anti-*β*2GPI complex alone group (*P* < 0 : 05; Figures 3(a)–3(d)). Consistent with the results of protein expressions, the addition of CQ in the oxLDL/*β*2GPI/anti-*β*2GPI complex treated group significantly increased the punctate staining of LC3 (Figures 3(c) and 3(d)). Taken together, these data supported that oxLDL/*β*2GPI/anti-*β*2GPI complex could block the autophagic flux in endothelial cells.

### 3.3. Effects of oxLDL/*β*2GPI/anti-*β*2GPI Complex on the Activation of PI3K/AKT/mTOR and eNOS in Endothelial Cells

**Figure 4:**
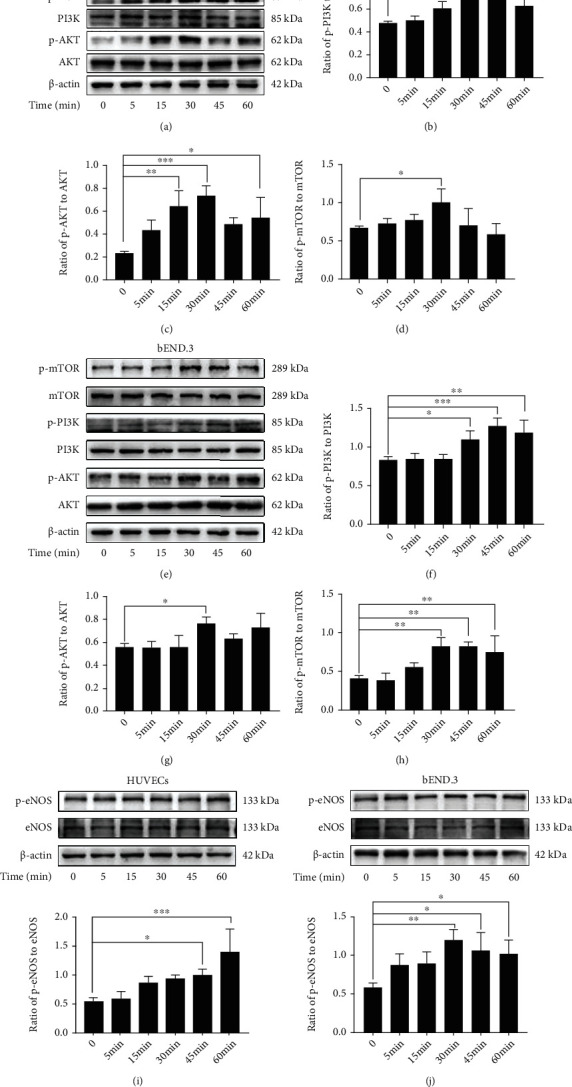
The phosphorylation of PI3K/AKT/mTOR and eNOS induced by the oxLDL/*β*2GPI/anti-*β*2GPI complex in endothelial cells. HUVECs and bEnd.3 cells were treated with oxLDL/*β*2GPI/anti-*β*2GPI complex for 5, 15, 30, 45, and 60 min. Western blotting analysis of p-PI3K, PI3K, p-AKT, AKT, p-mTOR, and mTOR in HUVECs (a) and bEnd.3 cells (e). Quantification of the ratio of p-AMPK/PI3K (b), p-AKT/AKT (c), and p-mTOR/mTOR (d) in HUVECs. Quantification of the ratio of p-AMPK/PI3K (f), p-AKT/AKT (g), and p-mTOR/mTOR (h) in bEnd.3 cells. Western blotting analysis and quantification for p-eNOS/eNOS expression in HUVECs (i) and bEnd.3 cells (j). ^∗^*P* < 0.05, ^∗∗^*P* < 0.01, and ^∗∗∗^*P* < 0.001 indicate statistically significant differences. ns: nonsignificant differences. All values are denoted as means ± SD from three independent experiments (*n* = 3), and a representative blot was shown.

We further examined the levels of PI3K/AKT/mTOR and eNOS that could negatively regulate autophagy [19, 37]. HUVECs and bEnd.3 cells were treated with oxLDL/*β*2GPI/anti-*β*2GPI complex, and then the expressions of PI3K/AKT/mTOR signaling pathway proteins (p-PI3K, PI3K, p-AKT, AKT, p-mTOR, and mTOR) and eNOS pathway proteins (p-eNOS and eNOS) were examined by western blotting analysis at 0, 5, 15, 30, 45, and 60 min (Figure 4). We found that the expression of p-PI3K was upregulated at 30 min after treatment with oxLDL/*β*2GPI/anti-*β*2GPI complex, which peaked at 45 min (*P* < 0 : 05; Figures 4(b) and 4(f)), while the expression of p-AKT was upregulated at 15 min (*P* < 0 : 05; Figure 4(c)) or 30 min (*P* < 0 : 05; Figure 4(g)), and the highest value was also observed at 30 min. The expression of p-mTOR was increased and peaked at 30 min after the oxLDL/*β*2GPI/anti-*β*2GPI complex stimulation (*P* < 0 : 05; Figures 4(d) and 4(h)). Meanwhile, the expression of p-eNOS was increased at 45 min in HUVECs (*P* < 0 : 05; Figure 4(i)) or 30 min in bEnd.3 cells (*P* < 0 : 01; Figure 4(j)). Moreover, we found that AZD5363 pretreatment significantly inhibited the expression of p-eNOS by suppressing AKT (*P* < 0 : 01; Figure S2), indicating the interaction between AKT and eNOS.

### 3.4. OxLDL/*β*2GPI/anti-*β*2GPI Complex Induces Autophagy Deficiency in PI3K/AKT/mTOR and eNOS Dependent Manner in Endothelial Cells

**Figure 5:**
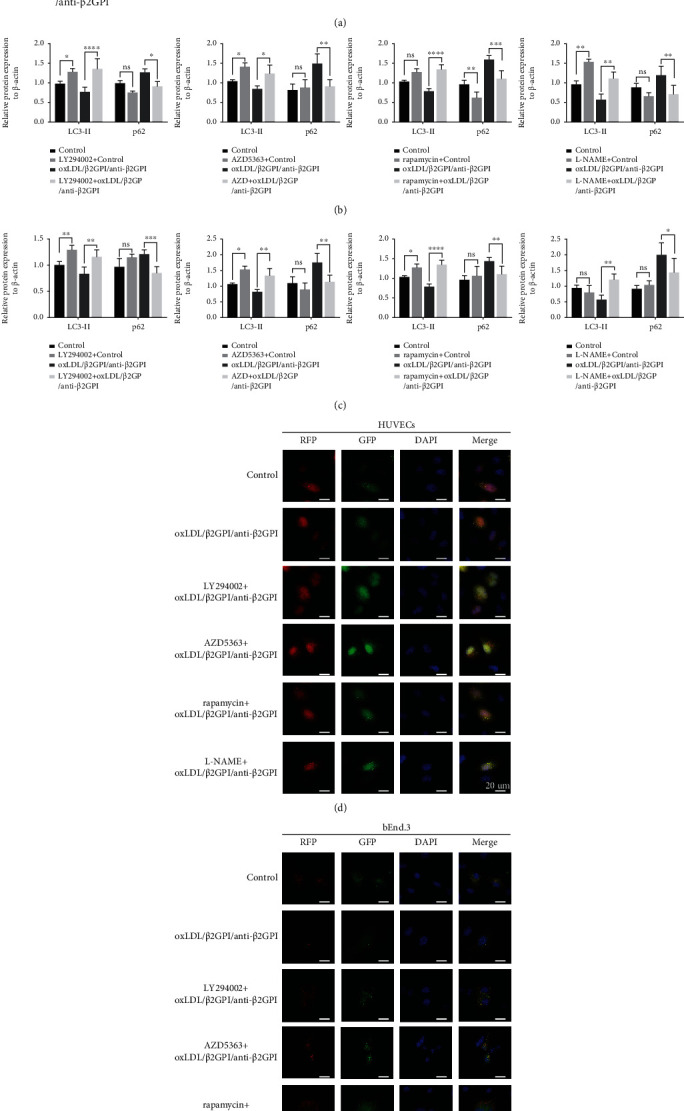
The regulation of PI3K/AKT/mTOR and eNOS signaling pathways in oxLDL/*β*2GPI/anti-*β*2GPI complex-mediated endothelial autophagy. HUVECs and bEnd.3 cells were incubated with oxLDL/*β*2GPI/anti-*β*2GPI complex for 24 h. LY294002 (10 *μ*M), AZD5363 (1 *μ*M), rapamycin (1 *μ*M), or L-NAME (100 *μ*M) were added 4 h before oxLDL/*β*2GPI/anti-*β*2GPI complex treatment. Western blotting analysis of p62 and LC3-II in HUVECs and bEnd.3 cells (a). Quantification of p62 and LC3-II in HUVECs (b) and bEnd.3 cells (c). Representative images (magnification, ×600) of RFP-GFP-LC3 puncta in HUVECs (d) and bEnd.3 cells (e). Yellow puncta (RFP^+^ and GFP^+^) represents autophagosomes, and red puncta (RFP^+^ and GFP^−^) represents autolysosomes. Scale bar: 20 *μ*m. ^∗^*P* < 0.05, ^∗∗^*P* < 0.01, ^∗∗∗^*P* < 0.001, and ^∗∗∗∗^*P* < 0.0001 indicate statistically significant differences. ns: nonsignificant differences. All values are denoted as means ± SD from three independent experiments (*n* = 3), and a representative blot/image was shown.

We investigated whether the signaling pathways explored above were involved in the effects of oxLDL/*β*2GPI/anti-*β*2GPI complex on suppressing autophagy of endothelial cells. We pretreated the cells with specific inhibitors of PI3K (LY294002), AKT (AZD5363), mTOR (rapamycin), or eNOS (L-NAME) and examined the effects of LY294002, AZD5363, rapamycin, or L-NAME on the expressions of autophagic proteins and the aggregation of LC3 puncta. We found that LY294002, AZD5363, rapamycin, and L-NAME pretreatment could significantly upregulate the expression of LC3-II, while downregulating the expression of p62 compared with the oxLDL/*β*2GPI/anti-*β*2GPI complex alone group (*P* < 0 : 05; Figures 5(b) and 5(c)). Additionally, the aggregation of LC3 puncta in the oxLDL/*β*2GPI/anti-*β*2GPI complex group was notably increased after pretreatment with LY294002, AZD5363, rapamycin, and L-NAME (Figures 5(d) and 5(e)), indicating that inhibition of PI3K/AKT/mTOR and eNOS pathways reversed the suppressive effects of oxLDL/*β*2GPI/anti-*β*2GPI complex on the autophagy.

### 3.5. Activation of Endothelial Autophagy Decreases the Expressions of oxLDL/*β*2GPI/anti-*β*2GPI Complex-Induced Inflammatory Cytokines in Endothelial Cells

**Figure 6:**
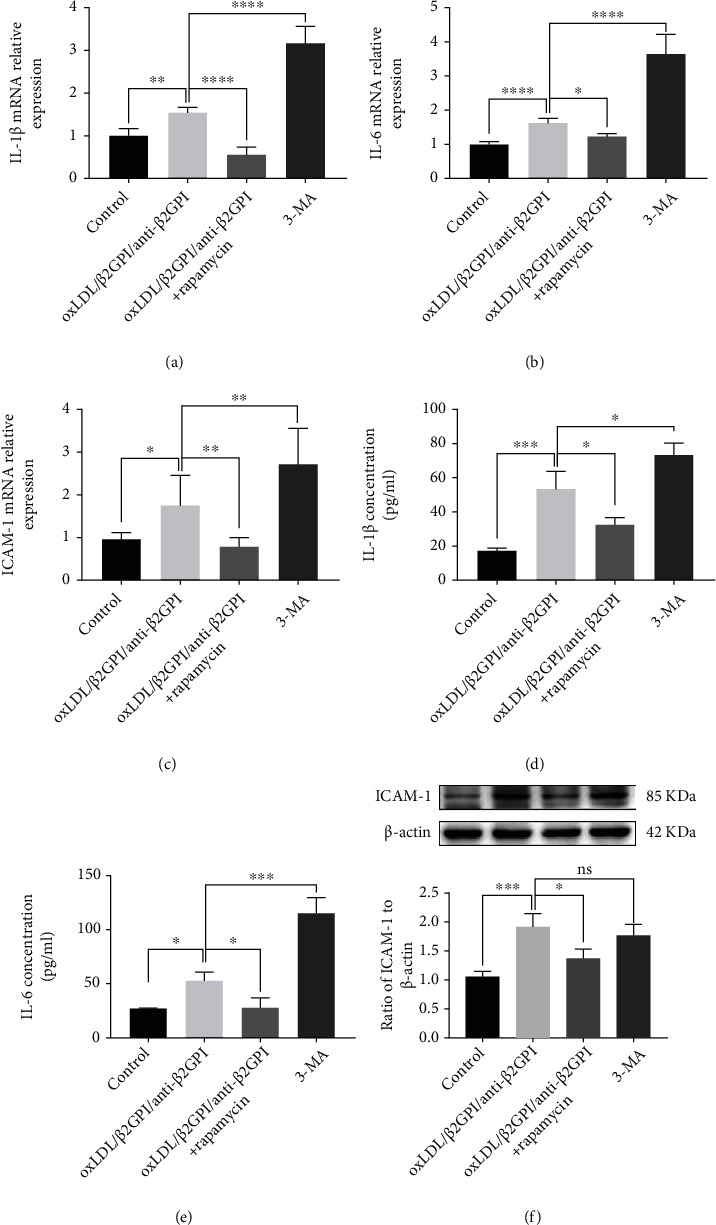
Activation of autophagy decreases oxLDL/*β*2GPI/anti-*β*2GPI complex-induced expressions of endothelial inflammatory cytokines. HUVECs were incubated with oxLDL/*β*2GPI/anti-*β*2GPI complex, oxLDL/*β*2GPI/anti-*β*2GPI complex + rapamycin (1 *μ*M, autophagy activator), and 3-MA (5 mM, autophagy inhibitor) for 6 h for mRNA detection and 24 h for protein detection. The expressions of IL-1*β* (a), IL-6 (b), and ICAM-1 at the mRNA level (c). Protein secretion of IL-1*β* (d) and IL-6 (e) was quantified in the cell supernatant by ELISA. ICAM-1 expression was detected using western blotting analysis (f). ^∗^*P* < 0.05, ^∗∗^*P* < 0.01, ^∗∗∗^*P* < 0.001, and ^∗∗∗∗^*P* < 0.0001 indicate statistically significant differences. ns: nonsignificant differences. All values are denoted as means ± SD from five independent experiments (*n* = 5).

Our previous study showed that oxLDL/*β*2GPI/anti-*β*2GPI complex is involved in the endothelial inflammatory response by promoting the expressions of various inflammatory cytokines [35]. In the present study, we further investigated whether oxLDL/*β*2GPI/anti-*β*2GPI complex-induced .autophagy suppression was associated with endothelial inflammation by using autophagy activator rapamycin and autophagy inhibitor 3-MA. Consistent with previous studies [38, 39], our results showed that the expression of LC3-II (Figure S3(a)) and the aggregation of LC3 puncta (Figure S3(b)) were increased after the treatment with rapamycin (1 *μ*M), while it was decreased after the treatment with 3-MA (5 mM) in HUVECs. We found that rapamycin treatment downregulated the expressions of IL-1*β*, IL-6, and ICAM-1 at the mRNA level compared with the oxLDL/*β*2GPI/anti-*β*2GPI complex alone group (Figures 6(a)–6(c)). Meanwhile, 3-MA significantly increased the expressions of these inflammatory cytokines compared with the oxLDL/*β*2GPI/anti-*β*2GPI complex alone group (Figures 6(a)–6(c)). Moreover, the secretion of IL-1*β* and IL-6 and the expression of ICAM-1 followed a similar trend (Figures 6(d)–6(f)).

### 3.6. Activation of Endothelial Autophagy Prevents oxLDL/*β*2GPI/anti-*β*2GPI Complex-Induced Oxidative Stress in Endothelial Cells

**Figure 7:**
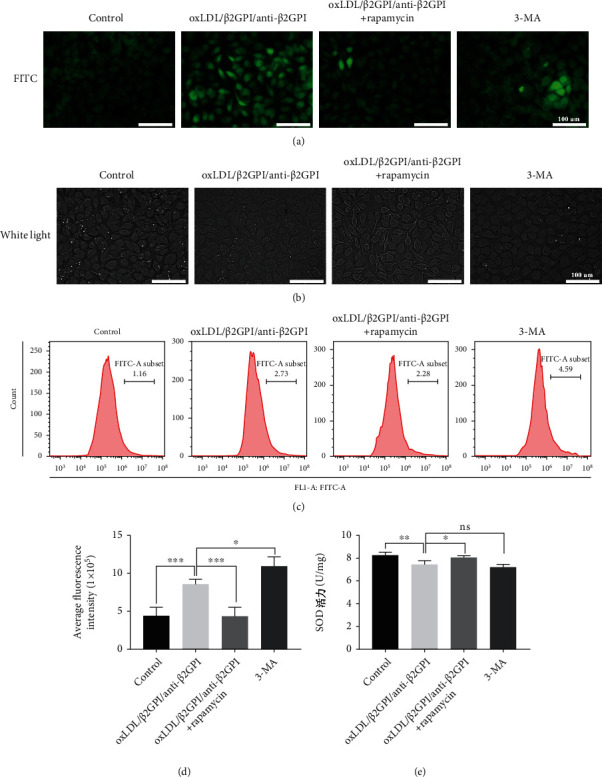
Activation of autophagy reduces oxLDL/*β*2GPI/anti-*β*2GPI complex-induced endothelial oxidative stress. HUVECs were incubated with oxLDL/*β*2GPI/anti-*β*2GPI complex, oxLDL/*β*2GPI/anti-*β*2GPI complex + rapamycin (1 *μ*M, autophagy activator), and 3-MA (5 mM, autophagy inhibitor) for 24 h. Representative images (magnification, ×200) of intracellular ROS production indicated by a fluorescent probe (DCFH-DA) (a). Cell morphology was observed by light microscopy (magnification, ×200) (b). ROS generation was detected by flow cytometry (c). Quantification of ROS fluorescence intensity (d). SOD activity was measured by the SOD assay kit (e). Scale bar: 100 *μ*m. ^∗^*P* < 0.05, ^∗∗^*P* < 0.01, ^∗∗∗^*P* < 0.001, and ^∗∗∗∗^*P* < 0.0001 indicate statistically significant differences. ns: nonsignificant differences. All values are denoted as means ± SD from three independent experiments (*n* = 3), and a representative image was shown.

Oxidative stress due to ROS accumulation has been critically linked to endothelial dysfunction, leading to the progression of AS [40]. Therefore, we detected the generation of ROS using the ROS detection dye DCFH-DA. Fluorescence microscopy showed that the production of ROS was markedly increased in oxLDL/*β*2GPI/anti-*β*2GPI complex-exposed cells, while the addition of rapamycin abolished the inductive effect of oxLDL/*β*2GPI/anti-*β*2GPI complex on ROS production (Figure 7(a)). The accumulation of ROS in the cells treated with autophagy inhibitor 3-MA was increased compared with the control group but not significantly changed compared with the oxLDL/*β*2GPI/anti-*β*2GPI complex alone treatment group (Figure 7(a)). We further confirmed these effects using flow cytometry and found that rapamycin could restore the promotion effects of oxLDL/*β*2GPI/anti-*β*2GPI complex-impaired autophagy on ROS production (Figures 7(c) and 7(d)). Meanwhile, the cells in the oxLDL/*β*2GPI/anti-*β*2GPI complex group and 3-MA group showed irregular shape with obscure borders, while the morphology of the cells was intact with a clear boundary after cotreatment with rapamycin and oxLDL/*β*2GPI/anti-*β*2GPI complex (Figure 7(b)). Moreover, we detected the generation of SOD. OxLDL/*β*2GPI/anti-*β*2GPI complex significantly decreased the SOD activity compared with the control group, while the addition of rapamycin reversed the effect of oxLDL/*β*2GPI/anti-*β*2GPI complex on SOD activity (Figure 7(e)). Besides, the SOD activity in the 3-MA group was decreased compared with the control group, and it showed no significant difference compared with the oxLDL/*β*2GPI/anti-*β*2GPI complex alone group (Figure 7(e)).

### 3.7. Activation of Endothelial Autophagy Suppresses oxLDL/*β*2GPI/anti-*β*2GPI Complex-Induced Endothelial Apoptosis

**Figure 8:**
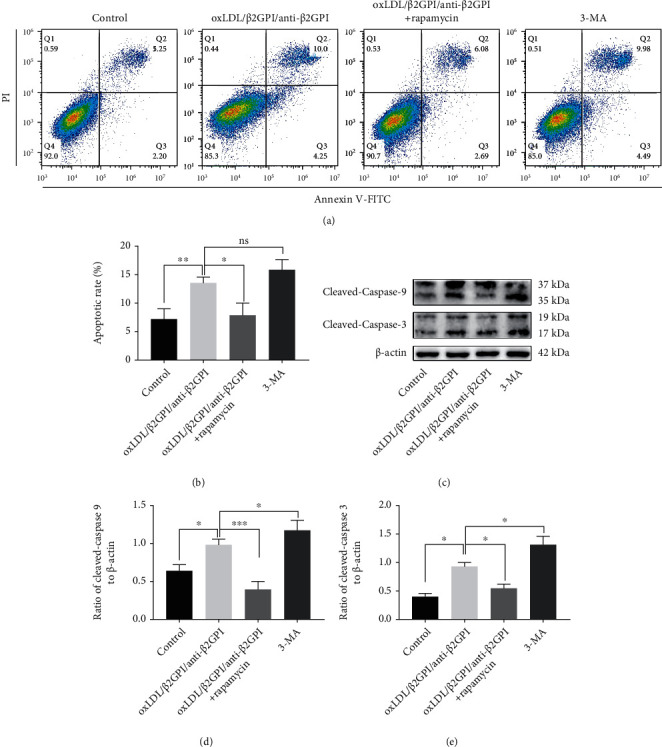
Activation of autophagy attenuates oxLDL/*β*2GPI/anti-*β*2GPI complex-induced endothelial apoptosis. HUVECs were incubated with oxLDL/*β*2GPI/anti-*β*2GPI complex, oxLDL/*β*2GPI/anti-*β*2GPI complex + rapamycin (1 *μ*M, autophagy activator), and 3-MA (5 mM, autophagy inhibitor) for 24 h. Representative images of apoptotic cells determined using flow cytometry (a). Quantitative statistics of the apoptotic rate (b). Western blotting analysis of cleaved caspase-3 and cleaved caspase-9 (c). Quantification of cleaved caspase-3 (d) and cleaved caspase-9 (e). ^∗^*P* < 0.05, ^∗∗^*P* < 0.01, and ^∗∗∗^*P* < 0.001 indicate statistically significant differences. ns: nonsignificant differences. All values are denoted as means ± SD from three independent experiments (*n* = 3), and a representative blot was shown.

Flow cytometry and western blotting analysis showed that oxLDL/*β*2GPI/anti-*β*2GPI complex induced the apoptosis of HUVECs (Figure 8), which was consistent with a previous study [41]. In parallel, we investigated the effect of autophagic induction on oxLDL/*β*2GPI/anti-*β*2GPI complex-induced apoptosis. We found that cotreatment of oxLDL/*β*2GPI/anti-*β*2GPI complex and rapamycin significantly suppressed the apoptosis of HUVECs compared with the oxLDL/*β*2GPI/anti-*β*2GPI complex alone group, while the 3-MA treatment group showed no obvious changes (Figures 8(a) and 8(b)). This finding was further supported by western blotting analysis, evidenced by a remarkable downregulation of cleaved caspase-3 and cleaved caspase-9 at the protein level in the cells treated with both oxLDL/*β*2GPI/anti-*β*2GPI complex and rapamycin (Figures 8(c)–8(e)). Notably, the expressions of cleaved caspase-3 and cleaved caspase-9 at the protein level in the 3-MA group were further increased compared with cells treated with oxLDL/*β*2GPI/anti-*β*2GPI complex alone (Figures 8(c)–8(e)), indicating that inhibition of autophagy could aggravate apoptosis.

## 4. Discussion

Previous studies have demonstrated that autoimmune response is involved in the pathogenesis of AS by contributing to the acceleration of atherosclerotic progression [42]. As an important immune complex in AS patients with an APS background, oxLDL/*β*2GPI/anti-*β*2GPI complex has been reported to have proatherogenic effects on most cells involved in AS, promoting the expressions of endothelial inflammatory cytokines, vascular smooth muscle cell proliferation, and macrophage foam cell formation [35, 43, 44]. With the discovery of autophagy, increasing attention has been paid to the alterations of autophagic flux during AS development and its relationship with AS-associated cellular damage [5, 45, 46]. Excess or deficiency in endothelial autophagy can result in cell death and injury and contribute to the formation of atherosclerotic plaques [5, 47]. In the present study, we investigated how oxLDL/*β*2GPI/anti-*β*2GPI complex regulated autophagy and how the changes in autophagy modulated inflammation, oxidative stress, and apoptosis in endothelial cells.

Western blotting analysis, TEM, and mRFP-GFP-LC3 tandem reporter assay were used to evaluate the expressions of autophagy-related proteins and autophagic processes in endothelial cells. It has been well established that Beclin-1, LC3, and p62 are the key proteins of autophagy, and Beclin1 and LC3II are upregulated during the activation of autophagy, accompanied by a decline of p62 [48]. Our study found that oxLDL/*β*2GPI/anti-*β*2GPI complex decreased the expressions of LC3-II and Beclin1 and increased the accumulation of p62 protein, indicating that oxLDL/*β*2GPI/anti-*β*2GPI complex could inhibit endothelial autophagy. Results from TEM and tandem fluorescence reporter assay showed a decreased number of autophagosomes after treatment with oxLDL/*β*2GPI/anti-*β*2GPI complex, reflecting the suppression of autophagic activity. OxLDL has been implicated in endothelial dysfunction by inducing multiple functional modifications in vascular endothelial cells [49]. This study showed that direct exposure to oxLDL could markedly enhance autophagy in endothelial cells, which was consistent with previous reports [50, 51]. Besides, the inhibitory effects of the oxLDL/*β*2GPI/anti-*β*2GPI complex on endothelial autophagy were much stronger compared with other control groups, suggesting the important role of the oxLDL/*β*2GPI/anti-*β*2GPI complex in the progression of AS with an APS background.

Considering that autophagy is a dynamic process [52], we used CQ (lysosomal degradation inhibitor) to further investigate the effect of oxLDL/*β*2GPI/anti-*β*2GPI complex on autophagic flux. The addition of CQ increased the accumulation of LC3-II and p62 in both HUVECS and bEnd.3 cells after the treatment of oxLDL/*β*2GPI/anti-*β*2GPI complex, suggesting that the observed decrease in LC3-II and increase in p62 induced by oxLDL/*β*2GPI/anti-*β*2GPI complex were attributed to the reduced initiation of autophagosomes rather than increased degradation of autophagosomes. Our mRFP-GFP-LC3 tandem reporter assay also confirmed that oxLDL/*β*2GPI/anti-*β*2GPI complex decreased the endothelial autophagic flux by inhibiting the formation of autophagosomes.

The PI3K/AKT/mTOR signaling pathway is known as a classic negative regulator of autophagy, and PI3K-AKT-mTOR signaling activation can promote cell death via autophagy suppression [14, 53]. Besides the PI3K/AKT/mTOR signaling pathway, eNOS is also an important downstream target of AKT, and the AKT/eNOS signal transduction pathway is involved in regulating autophagy [16, 19]. In the present study, oxLDL/*β*2GPI/anti-*β*2GPI complex could induce the phosphorylation of PI3K, AKT, mTOR, and eNOS, which was consistent with its inhibitory effects on endothelial autophagy observed in our present work. The results indicated that the PI3K/AKT/mTOR and eNOS signaling pathways might participate in the autophagic process induced by oxLDL/*β*2GPI/anti-*β*2GPI complex. To further confirm this deduction, specific inhibitors of PI3K/AKT/mTOR and eNOS, LY294002, AZD5363, rapamycin, and L-NAME, were used. AKT suppression by AZD5363 pretreatment inhibited eNOS activation, which verified the previous conclusion that eNOS is a downstream target of AKT [19]. The increased p62 and decreased LC3-II protein expression, as well as the reduced autophagosomes induced by oxLDL/*β*2GPI/anti-*β*2GPI complex, were markedly attenuated by the inhibitors aforementioned, suggesting that oxLDL/*β*2GPI/anti-*β*2GPI complex could inhibit autophagy via activating PI3K/AKT/mTOR and AKT/eNOS signaling pathways in endothelial cells.

Endothelial cell dysfunction is related to increased endothelial cell inflammation, oxidative stress, and apoptosis, all of which are involved in vascular injury and atherosclerotic lesions [54]. It is generally known that autophagy can be pharmacologically induced by inhibiting mTOR with rapamycin, whereas 3-MA inhibits autophagy by targeting the class III PI3K involved in autophagosome formation [55]. IL-1*β*, IL-6, and ICAM-1 are important proinflammatory molecules, which play crucial roles in the preliminary inflammatory response and endothelial dysfunction [56]. Our results showed that rapamycin could partially reverse the upregulation of IL-1*β*, IL-6, and ICAM-1 induced by oxLDL/*β*2GPI/anti-*β*2GPI complex, while the 3-MA treatment resulted in increased expressions of inflammatory cytokines. These data suggested that oxLDL/*β*2GPI/anti-*β*2GPI complex-induced endothelial inflammation was associated with the inhibition of autophagy.

A previous study has also demonstrated that oxLDL/*β*2GPI/anti-*β*2GPI complex contributes to oxidative stress by increasing the ROS production in endothelial cells [41]. Oxidative stress is a chief cause of vascular endothelial damage, resulting in excessive ROS production and leading to endothelial injury via the inhibition of nitric oxide production [40, 57]. As increased ROS level is associated with cell dysfunction and reduced cell survival and SOD is an antioxidant enzyme that can neutralize ROS [58], we simultaneously tested the ROS production and SOD activity in HUVECs. We found that oxLDL/*β*2GPI/anti-*β*2GPI complex improved the SOD activity and lowered the production of ROS, which was consistent with a previous study [41]. This effect of oxLDL/*β*2GPI/anti-*β*2GPI complex on endothelial oxidative stress could be rescued by the addition of rapamycin, while the autophagy inhibitor 3-MA had a similar effect as oxLDL/*β*2GPI/anti-*β*2GPI complex. Notably, the cell boundaries were indistinct after treatment with oxLDL/*β*2GPI/anti-*β*2GPI complex or 3-MA, while the neatly arranged cells with clear boundaries were observed in oxLDL/*β*2GPI/anti-*β*2GPI complex group after the addition of rapamycin. Therefore, autophagy played an important role in mediating oxidative stress and maintaining the morphology of endothelial cells.

Moreover, apoptosis of endothelial cells is an important factor of vascular injury, which is directly related to the degree of endothelial damage [59]. In the present study, we observed that oxLDL/*β*2GPI/anti-*β*2GPI complex increased the number of apoptotic cells and enhanced the expressions of proapoptotic proteins. Simultaneously, suppressing autophagy by 3-MA resulted in increased apoptosis of HUVECs. In contrast, rapamycin could decrease the degree of apoptosis induced by oxLDL/*β*2GPI/anti-*β*2GPI complex. Our results implied that impaired autophagy induced by oxLDL/*β*2GPI/anti-*β*2GPI complex was linked to endothelial apoptosis. Nevertheless, our results suggested that impaired endothelial autophagy resulted in increased inflammation, oxidative stress, and apoptosis, which triggered vascular endothelial injury and led to the atherosclerotic lesion.

**Figure 9:**
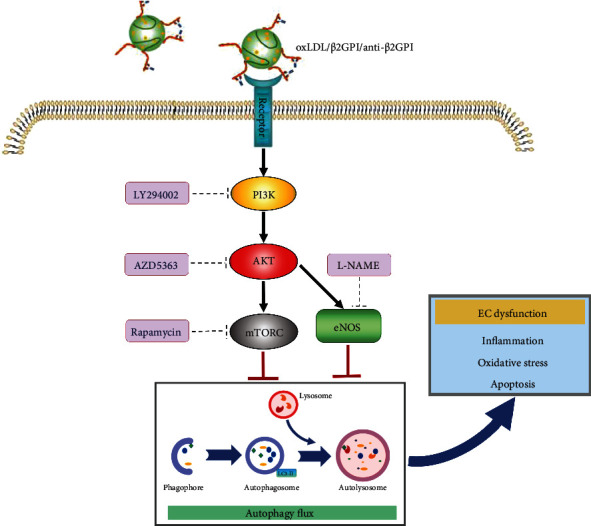
Proposed model for autophagy inhibition-promoted endothelial cell dysfunction induced by oxLDL/*β*2GPI/anti-*β*2GPI complex. OxLDL/*β*2GPI/anti-*β*2GPI complex, the circulating immune complex combined by oxLDL/*β*2GPI complex and anti-*β*2GPI, could suppress the autophagy process through activating both PI3K/AKT/mTOR and eNOS in endothelial cells. This inhibition would lead to elevated inflammation, oxidative stress, and apoptosis, which finally induced endothelial cell dysfunction. Abbreviations: oxLDL: oxidized low-density lipoprotein; *β*2GPI: *β*2-glycoprotein I; anti-*β*2GPI: anti-*β*2-glycoprotein I antibody; PI3K: phosphatidylinositol-3 kinase; AKT: serine/threonine kinase; mTOR: the kinase mammalian target of rapamycin; eNOS: endothelial nitric oxide synthase.

Collectively, our findings indicated that oxLDL/*β*2GPI/anti-*β*2GPI complex could inhibit the endothelial autophagy by PI3K/AKT/mTOR and eNOS signaling pathways, and the impaired autophagy further promoted inflammation, oxidative stress, and apoptosis in endothelial cells (Figure 9). Taken together, our findings provided valuable insights into the molecular mechanisms underlying endothelial injury and the pathogenesis of AS with an APS background.

## Figures and Tables

**Figure 1:**
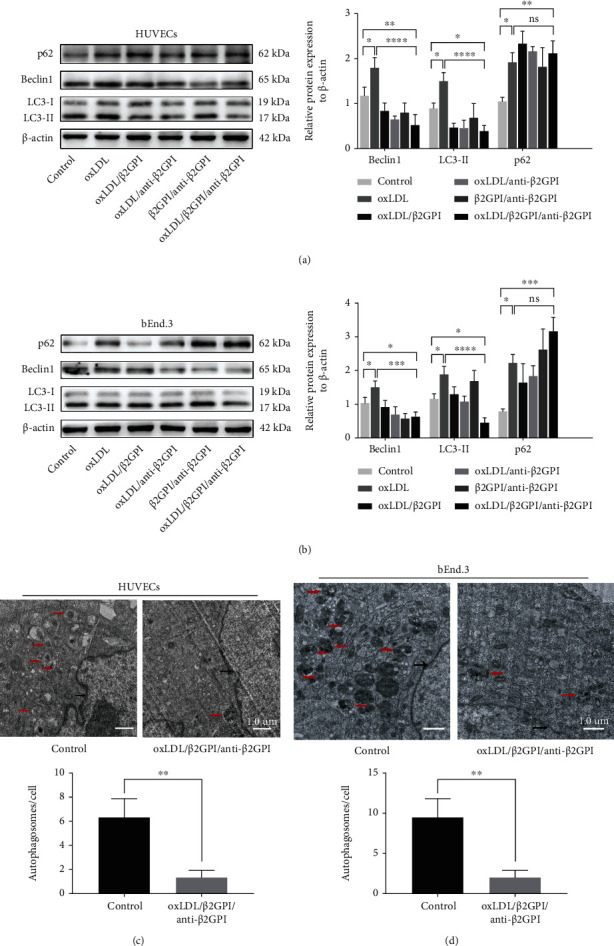
Protein expression analysis and TEM observation of the effect of oxLDL/*β*2GPI/anti-*β*2GPI complex on endothelial cell autophagy. HUVECs and bEnd.3 cells were incubated with oxLDL, oxLDL/*β*2GPI, oxLDL/anti-*β*2GPI, *β*2GPI/anti-*β*2GPI, and oxLDL/*β*2GPI/anti-*β*2GPI complex for 24 h. Western blotting analysis and quantification of p62, Beclin1, and LC3 in HUVECs (a) and bEnd.3 cells (b). Representative images (magnification, ×3000) and quantification from TEM showing the autophagy lysosome or autophagosomes in HUVECs (e) and bEnd.3 cells (f). The red arrows indicating autophagy lysosome or autophagosome and the black arrows indicating double nuclear membrane. Scale bar: 1.0 *μ*m. ^∗^*P* < 0.05, ^∗∗^*P* < 0.01, ^∗∗∗^*P* < 0.001, and ^∗∗∗∗^*P* < 0.0001 indicate statistically significant differences. ns: nonsignificant differences. All values are denoted as means ± SD from five independent experiments (*n* = 5), and a representative blot/image was shown.

## Data Availability

The data used to support the findings of this study are available from the corresponding author upon request.

## Supplementary Materials

Supplementary Materials Table S1: genes and sequence of primer pairs used for RT-qPCR. Figure S1: effects of oxLDL/*β*2GPI/anti-*β*2GPI complex on the expressions of autophagy-related proteins at different time points in HUVECs. Figure S2: identification of the effects of AZD5363 on eNOS activation in HUVECs. Figure S3: identification of the effects of rapamycin and 3-MA on endothelial cell autophagy.
