# Supplementary material for: Impaired Autophagy Induced by oxLDL/β2GPI/anti-β2GPI Complex through PI3K/AKT/mTOR and eNOS Signaling Pathways Contributes to Endothelial Cell Dysfunction
Source: Oxid Med Cell Longev. 2021 Jun 14;2021:6662225. doi: 10.1155/2021/6662225 (PMC8219424; doi:10.1155/2021/6662225)

**Supplementary tables**

**Table S1. Genes and sequence of primer pairs used for RT-qPCR.**

| Gene name | Sequence of primer pairs  (5’-3’) | Genbank  accession |
| --- | --- | --- |
| IL-1β (144 bp) | Forward: TCGCCAGTGAAATGATGGCT  Reverse: GGTCGGAGATTCGTAGCTGG | NM_000576.3 |
| IL-6 (150 bp) | Forward: TGCAATAACCACCCCTGACC  Reverse: ATTTGCCGAAGAGCCCTCAG | NM_001371096.1 |
| ICAM-1 (69 bp) | Forward: AGCTTCGTGTCCTGTATGGC  Reverse: TTTCTGGCCACGTCCAGTTT | NM_000201.3 |
| β-actin (265 bp) | Forward: CACGAAACTACCTTCAACTCC  Reverse: CATACTCCTGCTTGCTGATC | NM_001101.5 |

**Supplementary figure legends**

**Figure S1. Effects of oxLDL/β2GPI/anti-β2GPI complex on the expressions of autophagy-related proteins at different time points in HUVECs**

HUVECs were incubated in the presence or absence of oxLDL/β2GPI/anti-β2GPI complex for 6, 12, 24 and 36 h. Western blotting analysis of Beclin1, p62, and LC3 in HUVECs (a). Quantification of Beclin1 (b), p62 (c), and LC3-Ⅱ (d) in HUVECs. ^**^*P* < 0.01, ^***^*P* < 0.001 and ^****^*P* < 0.0001 indicate statistically significant differences. All values are denoted as means ± SD from three independent experiments (n = 3), and a representative blot was shown.

**Figure S2.** **Effects of AZD5363 on the activation of eNOS in HUVECs**

HUVECs were treated with/without AZD5363 (1 μM, AKT inhibitor) for 4 h and then incubated with oxLDL/β2GPI/anti-β2GPI complex for 24h. Western blotting analysis of p-eNOS and eNOS in HUVECs (a). Quantification of the ratio of p-eNOS/eNOS (b) in HUVECs. ^*^*P* < 0.05, ^**^*P* < 0.01. All values are denoted as means ± SD from three independent experiments (n = 3), and a representative blot was shown.

**Figure S3. Protein expression analysis and RFP-GFP-LC3 tandem fluorescent probe analysis of the effects of rapamycin and 3-MA on endothelial cell autophagy**

HUVECs were incubated with oxLDL/β2GPI/anti-β2GPI complex, rapamycin (1 μM, autophagy activator) and 3-MA (5 mM, autophagy inhibitor) for 24 h. Western blotting analysis of LC3 in HUVECs (a). Representative images (magnification, x600) of RFP-GFP-LC3 puncta in HUVECs (b). Yellow puncta (RFP+ and GFP+) represents autophagosomes, and red puncta (RFP+ and GFP-) represents autolysosomes. ^*^*P* < 0.05, ^**^*P* < 0.01. All values are denoted as means ± SD from three independent experiments (n = 3), and a representative blot/image was shown.

**Figure S1. Effects of oxLDL/β2GPI/anti-β2GPI complex on the expressions of autophagy-related proteins at different time points in HUVECs (related to Result 3.1)**


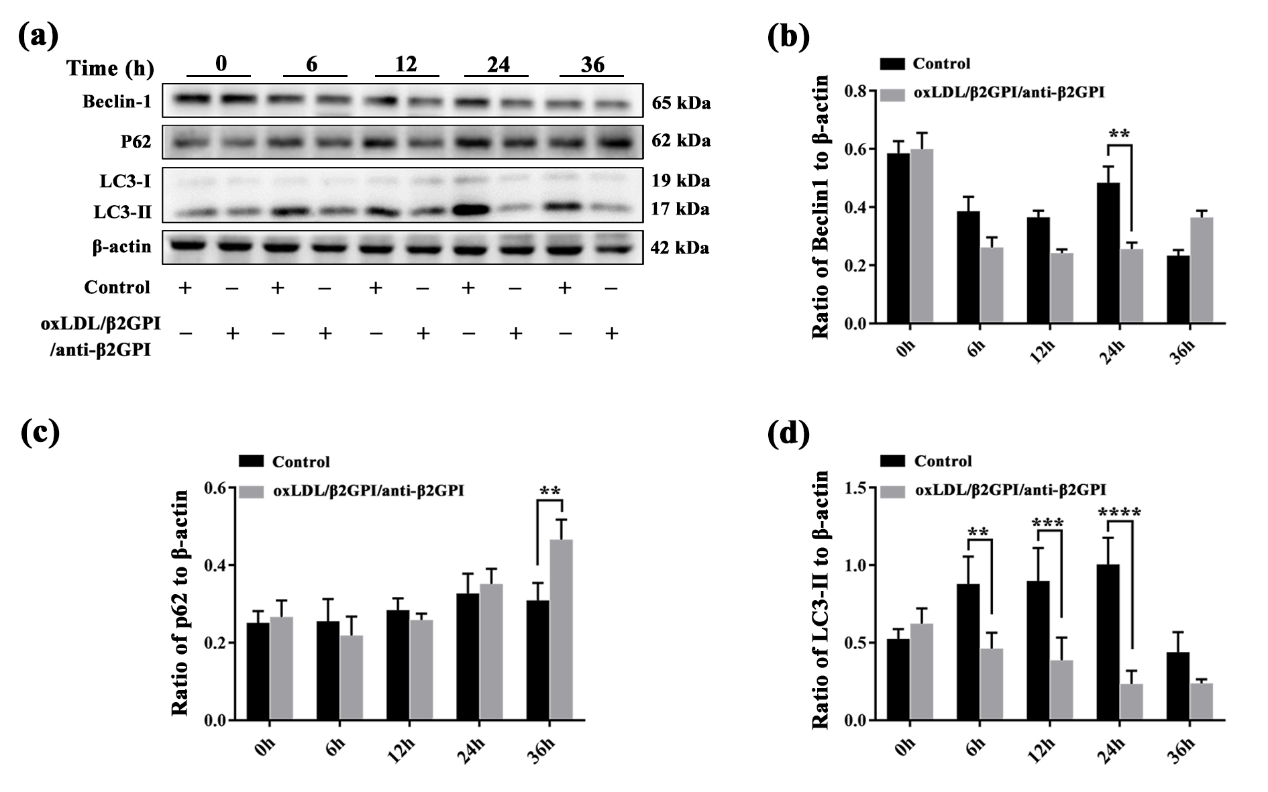


**Figure S2. Identification of the effects of AZD5363 on eNOS activation in HUVECs (related to Result 3.3)**


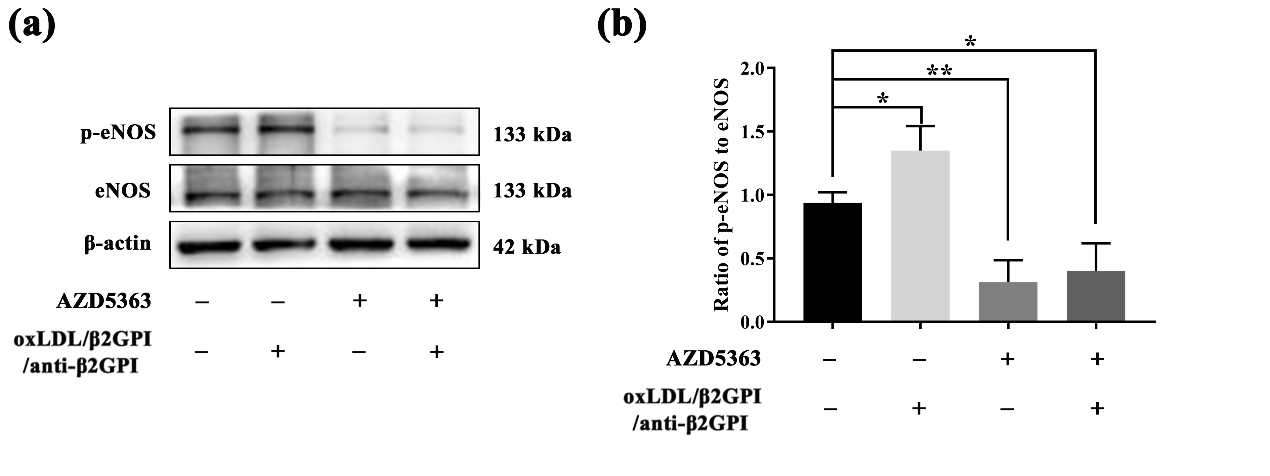


**Figure S3. Identification of the effects of Rapamycin and 3-MA on endothelial cell autophagy (related to Result 3.5)**


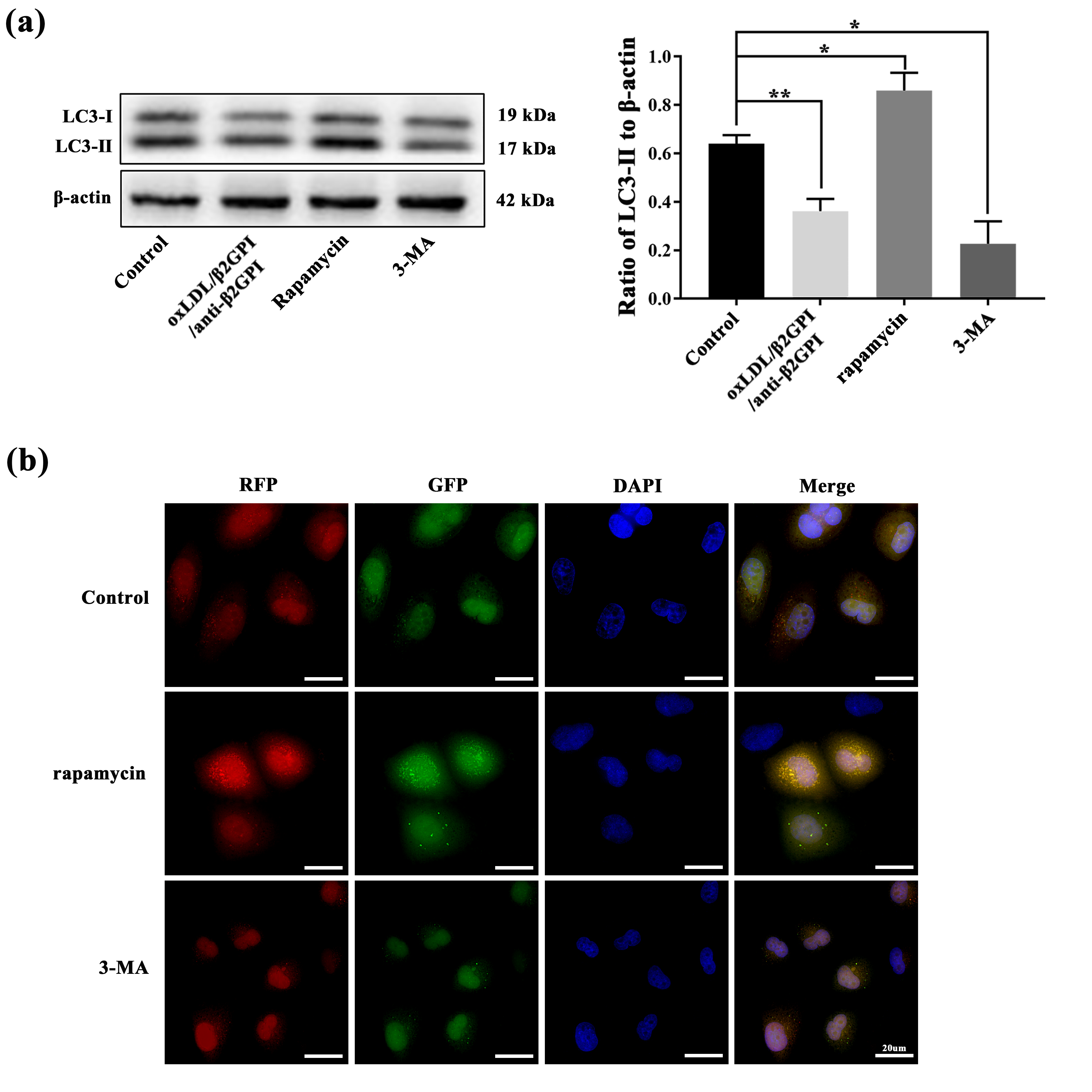

Supplement: Supplementary Materials — Table S1: genes and sequence of primer pairs used for RT-qPCR. Figure S1: effects of oxLDL/β2GPI/anti-β2GPI complex on the expressions of autophagy-related proteins at different time points in HUVECs. Figure S2: identification of the effects of AZD5363 on eNOS activation in HUVECs. Figure S3: identification of the effects of rapamycin and 3-MA on endothelial cell autophagy. [file 6662225.f1.docx]
